# Case Report: Simultaneous Resection of Bone Tumor and CAD/CAM Titanium Cranioplasty in Fronto-Orbital Region

**DOI:** 10.3389/fsurg.2021.718725

**Published:** 2021-10-27

**Authors:** Konstantin S. Yashin, Anton Yu. Ermolaev, Mikhail V. Ostapyuk, Marina A. Kutlaeva, Marina V. Rasteryaeva, Sergey G. Mlyavykh, Igor A. Medyanik

**Affiliations:** ^1^Department of Oncology and Neurosurgery, Institute of Traumatology and Orthopedics, Privolzhsky Research Medical University, Nizhny Novgorod, Russia; ^2^Department of Radiation Diagnostics, Institute of Traumatology and Orthopedics, Privolzhsky Research Medical University, Nizhny Novgorod, Russia; ^3^Institute of Traumatology and Orthopedics, Privolzhsky Research Medical University, Nizhny Novgorod, Russia

**Keywords:** CAD/CAM, cosmetic outcome, drilling template, single-step resection and reconstruction, skull bone tumors, titanium cranioplasty, custom-made implants, skull reconstruction

## Abstract

**Background:** Simultaneous resection of bone tumors in the fronto-naso-orbital region is a great challenge due to the need for adequate reconstruction of the facial skeleton. Pre-operative virtual planning of resection margins and the simultaneous fabrication of the cranioplasty using computer-aided design/computer-aided manufacturing (CAD/CAM) technology could allow combining the tumor resection and cosmetic restoration steps into a single procedure.

**Methods:** We present five consecutive cases of patients with bone tumors of the fronto-naso-orbital region. The indications for surgery included: (1) the presence of a major cosmetic defect; (2) progressive tumor growth. The histological examination revealed vascular malformation, hemangioma, and fibrous dysplasia in two cases. Tumor resection was performed with the help of a drilling template in form of a tumor. The computer-designed cranioplasty formed based on the non-involved side of the skull of the patient was manufactured. In one patient, the reconstruction was performed using two separate implants.

**Results:** The position of the implant fits in with pre-operative planning in two cases; in those cases, the additional trimming of the implant or bone defect was required. Good cosmetic outcomes were noted in all patients, and no complications occurred. No repeat surgery was necessary. The template has proved to have high application potential.

**Conclusion:** Simultaneous resection and CAD/CAM cranioplasty in the case of bone tumors in the fronto-orbital region is a promising technique with the aim of minimizing operation time and achieving a good esthetic outcome.

## Introduction

In patients with skull bone tumors, it was demonstrated that not only the oncological but also the cosmetic result has a significant influence on the long-term outcome (1, 2). It becomes more significant in the case of lesions in the fronto-orbital area, having an important functional and aesthetic role.

The adequate reconstruction of facial contours after craniofacial traumas is a great challenge due to the unique sophisticated spatial complexity of this region (3). A more challenging task is a restoration of facial symmetry during a one-step resection of lesions in the craniofacial region, following a skull reconstruction, where contour and shape may be difficult to achieve with intraoperative modeling of autogenous bone grafts, titanium, or another synthetic material (4). Thus, the two-step procedure has been proposed: a pre-operative-performed patient specific implant (PSI) is inserted in a second surgery. However, two-step-technology determinates to weigh the pros and cons, with calculation, the risk of surgery-associated complications in relation to the profit for the patient, which, most of all, is a cosmetic improvement.

To improve the precision of surgical resection and reconstruction procedures, computer pre-operative planning and intraoperative navigation can be used (4–6). The next step is performing a one-step template-assisted resection of bone tumor following a cranioplasty with the PSI based on the virtual planning of the resection margins, which has already been reported in the cases of fibrous dysplasia and intraosseous skull base meningiomas (7, 8).

In this article, we present the results of simultaneous resection and computer-aided design/computer-aided manufacturing (CAD/CAM) titanium cranioplasty in four consecutive treating patients with bone tumors in the fronto-orbital region. This technology looks most promising in this group of patients, with the aim of minimizing operation time and achieving a good esthetic outcome.

## Methods

### Patients

Four surgical cases with a pathology involving the fronto-orbital region were included in the study. The general characteristics of patients are summarized in Table 1. The indications for surgery were: (1) the presence of a major cosmetic defect; (2) progressive tumor growth. The histological examination revealed vascular malformation, hemangioma, and fibrous dysplasia in two cases. The details of the surgical procedure were discussed before hospitalization, and informed consent was obtained from the patients. Written informed consent was obtained from the individuals for the publication of any potentially identifiable images or data included in this article. This study was carried out in accordance with the recommendations of the World Medical Association's Declaration of Helsinki. The protocol was approved by the Ethical Committee of the Privolzhsky Research Medical University of the Ministry of Health of the Russian Federation. The post-operative follow-up was up to 5 years.

**Table 1:** Patient profiles, surgery, and results.

**Patient no**.	**Age/gender**	**Diagnosis**	**Indication for surgery**	**Using template**	**Number of implants**
1	36, F	Fibrous dysplasia	Growth	No	Two
2	35, F	Vascular malformation	Cosmetic	Yes	One
3	44, F	Fibrous dysplasia	Cosmetic	Yes	One
4	40, F	Hemangioma	Cosmetic/growth	Yes	One

### Pre-operative Planning and Manufacturing of the Cranioplasty

**Figure 1:**
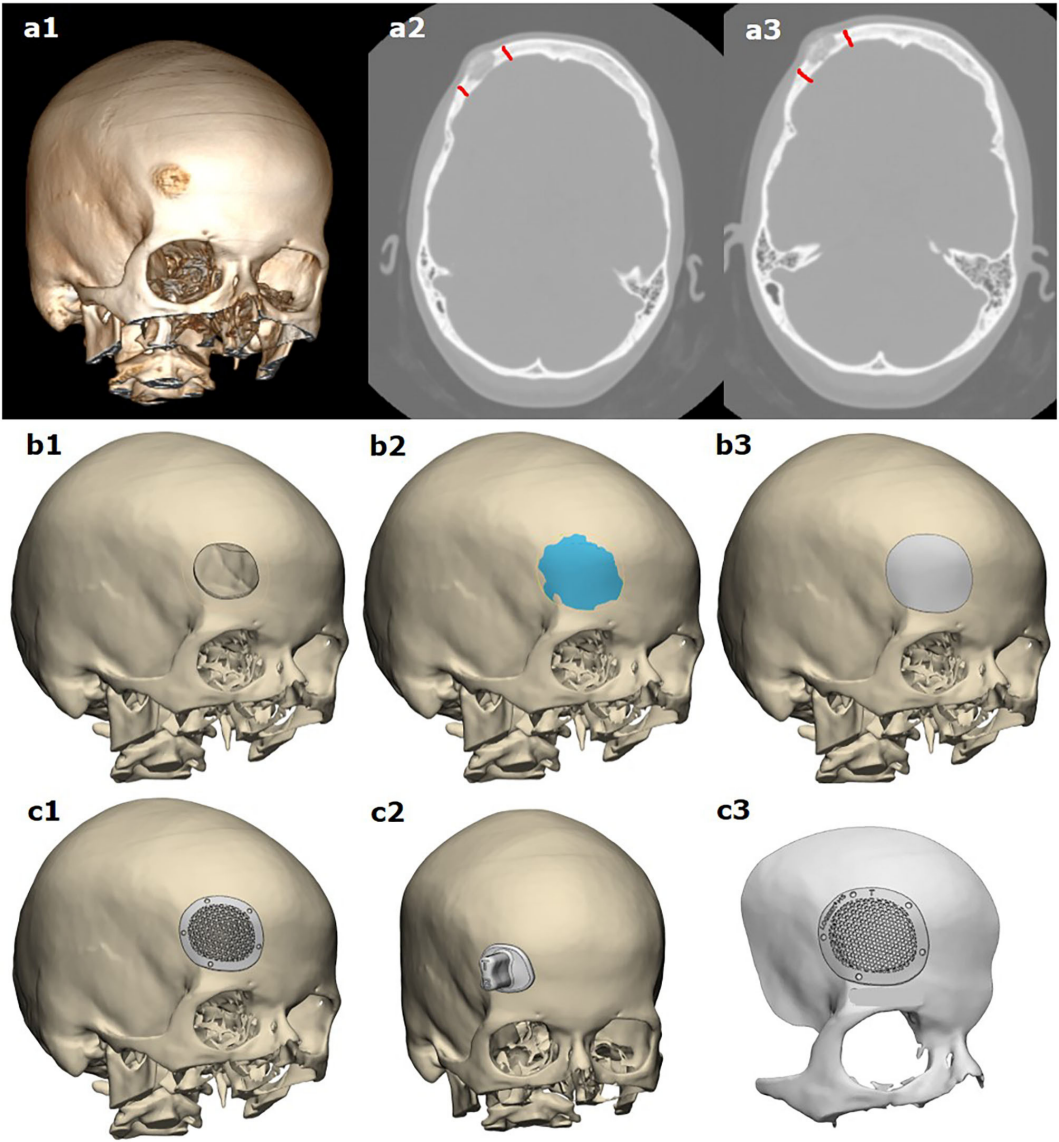
Modeling of template-assisted resection of bone tumor, following cranioplasty based on virtual planning of the resection margins: **(a1–a3)** based on CT scans, planning of resection margins made by the surgeon; **(b1–b3)** 3D modeling of tumor resection and the anatomical shape of the resected part of the bone; **(c1, c2)** 3D modeling of the implant and resection template; **(c3)** a pre-operative planning template.

Resection planning and manufacturing of the cranioplasty in one session were planned in several steps in collaboration with the specialists of two manufacturing companies: Endoprint Innovation and Technology Company (Endoprint ITK LLC, Moscow, Russian Federation) and LOGEEKS DM (Logix LLC, Novosibirsk, Russian Federation). Using 0.5-mm computer tomography (CT) scan slices, the surgeon drew a digital resection line (Figures 1a1–a3). Based on this resection line, the reconstruction of future defect margins was performed using CAD software (Figures 1b1–b3). The skull surface in the resection area is generated as a CAD surface by mirroring the opposite side of the skull.

Corresponding to this CAD surface, the computer modeling of the implant was performed, and screw holes over the non-affected bone are integrated (Figures 1c1–c3). In three cases, a resection template was constructed for the defect using the identical geometric data of the resected bone with tumor. When both the implant and the template are constructed completely, the design has to be released by the neurosurgeons.

Based on the results of virtual surgical planning of the proposed skull defect after lesion resection, the implants were growing by the 3D metal printing method—direct metal laser sintering (DMLS). After that, during manufacturing, the implants go through stages such as sandblasting, ultrasonic washing, and disinfection.

### Surgical Procedure

**Figure 2:**
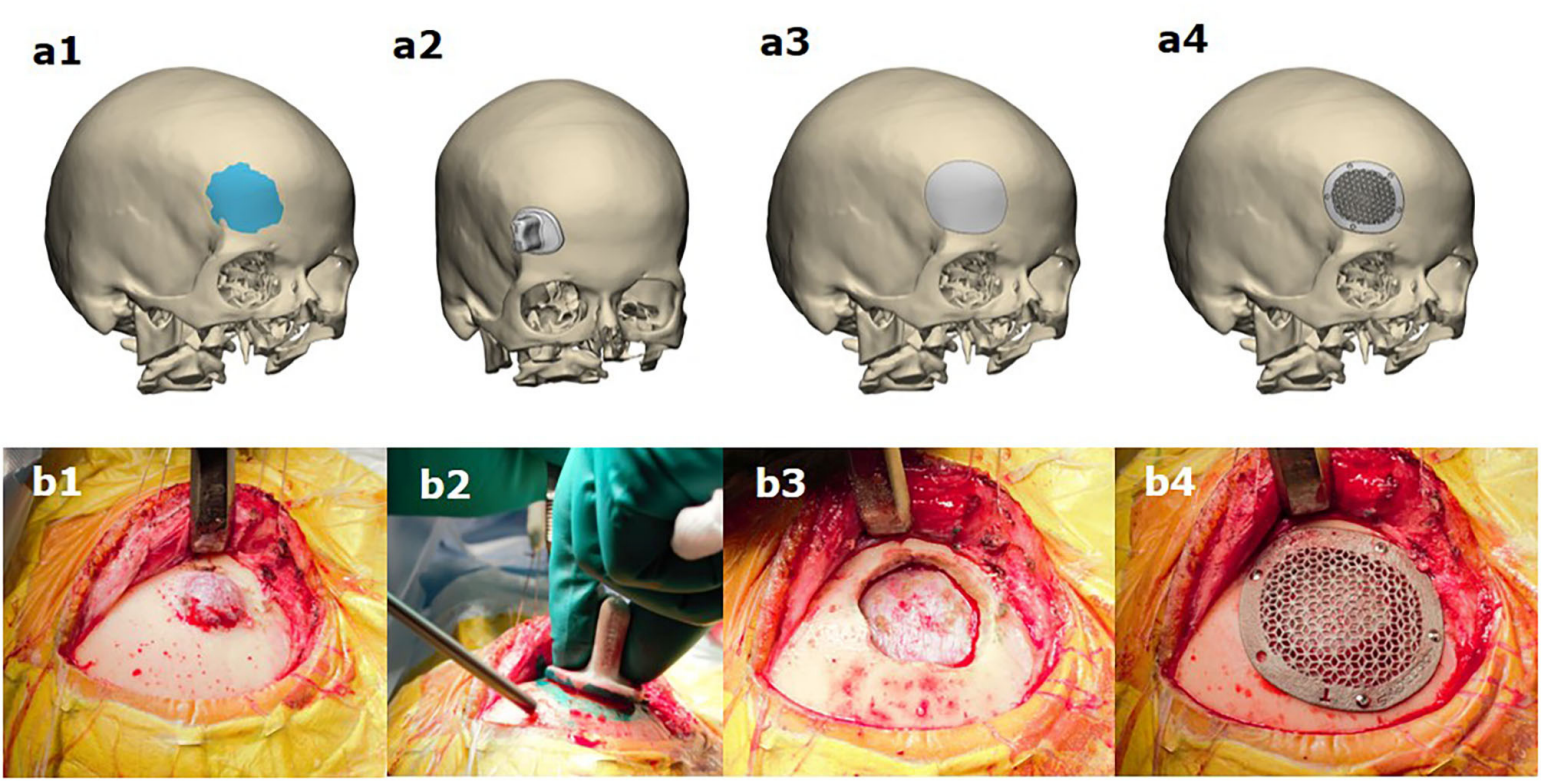
Simultaneous resection of bone tumor and titanium cranioplasty in patients with a lesion in the fronto-orbital region **(b1–b4)** according to pre-operative virtual planning of resection margins **(a1)**, using a drilling template **(a2)** and the simultaneous fabrication of the cranioplasty **(a3,a4)**.

The procedures were performed under general anesthesia according to the pre-operative virtual plan (Figure 2). The part of the cranium that matched the interior surface of the template was identified, and the osteotomy line was drawn along the edge of the template. An osteotomy was performed along the line, resulting in the removal of the tumor with the bone. In no one case, the reconstruction of the dura was necessary to perform. The implant was then placed in the skull defect and fixed with screws. In one patient, the reconstruction was performed using two separate implants.

## Results

**Figure 3:**
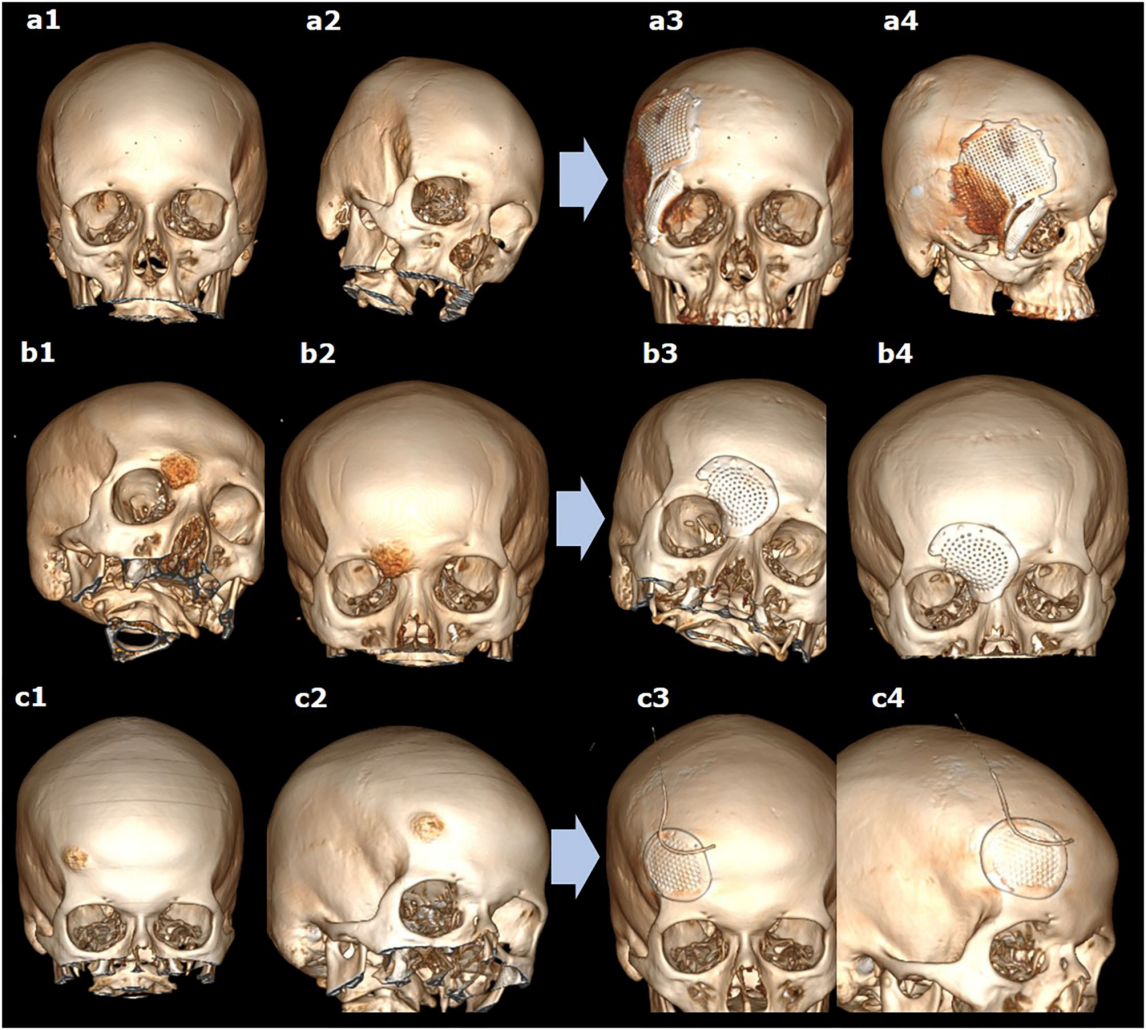
The 3D reconstructions of CT scans before **(a1–c1,a2–c2)** and after **(a3–c3,a4–c4)** simultaneous resection of bone tumor and titanium cranioplasty in different cases of lesions in the fronto-orbital region, using two **(a1–a4)** or one **(b1–b4,c1–c4)** implants.

The position of the implant fits in with pre-operative planning. In three cases, the complete resection confirmed on post-operative CT scans of the lesion was achieved. In one case of fibrous dysplasia, the subtotal resection was performed. On post-operative CT scans, no continued growth was detected at up to 5-year follow-up. Good cosmetic outcomes were noted in all patients, and no complications occurred (Figures 3a1–c4). No repeat surgery was necessary. The usage of the template was highly practicable. Perioperative trimming of the implant or bone defect was not required in two patients. In one patient, the implant was additionally modeling during the procedure, preserving the branches of nervus supraorbitalis (Figures 3b1–b4). The template used was not possible in the case of severe fibrous dysplasia due to the shape and the size of resection (Figures 3a1–a4). Therefore, additional bone drilling was required for adequate implant positioning.

## Discussion

Individual CAD/CAM cranioplasty for the reconstruction of extensive and complex skull defects has been a recently actively developing field of neurosurgery and craniomaxillofacial surgery (9, 10). The patient-specific reconstruction of a bone flap becomes a preferred method in clinical practice due to a favorable functional and cosmetic outcome compared with the other methods. Pre-operative computer modeling and using CAD/CAM technologies have expanded the possibilities of surgery for achieving good cosmetic and functional results in the treatment of bone tumors. It allows abandoning the conventional techniques of reconstruction or two-step procedures that are particularly important in the case of tumor resection in the fronto-orbital region.

The one-step template-assisted resection of bone tumor following cranioplasty has been already reported in the cases of including fibrous dysplasia and intraosseous meningiomas (7, 8, 11). This method looks promising for intraosseous tumors compared with other modalities, such as intraoperatively modeling an artificial bone flap without a template (1, 12) or manufacturing the custom-made implant based on the stereolithographic model (13). It has several advantages over other reported methods: (1) pre-operative time and costs benefits, all steps of modeling are performed virtually, so no additional production steps are needed; (2) operation time, the using of the template allows to perform resection and cranioplasty fast and precisely; (3) the best available cosmetic and functional result for resection of fronto-orbital region tumors. This corresponds to the results of the application of CAD/CAM custom implants in patients with skull defects with satisfactory aesthetic results, technically simplicity, reducing surgery times, and surgical blood loss (14).

In most cases of bone tumor location, the skull reconstruction would have not required a patient specific implant since a standard titanium cranioplasty could have been easily adapted. However, we believe that in the case of the location of the tumor in the bones forming the facial region of the skull, using PSI will be the method of choice, achieving the best congruence and cosmetic results. Moreover, using the customized template can help with the resection and reduce the time of the procedure even when the reconstruction does not require a PSI. Using a template as an alternative to navigation systems, especially in the case of the poorly visible extracranial component, is controversial. It is obvious that navigation can be very useful for contouring tumors (6, 15). Also, the specific approach combining “mirroring” virtual computational planning with intraoperative-guided surgical navigation was suggested (5). We believe that the combination of navigation and a drilled template provides the best results since navigation can be used for the planning of the site and the size of skin resection and clarification of the resection margins.

For cranioplasty, a variety of alloplastic biomaterials can be used, including standard hydroxyapatite (HA), polymethylmethacrylate (PMMA), polyetheretherketone (PEEK), titanium, and actively developing new substances (16–18). Each has its own advantages and disadvantages; most of them are suitable for CAD/CAM pre-fabrication, and all of them are potentially biologically toxic, with the likely exception of hydroxyapatite (14). For simultaneous tumor resection and cranioplasty, titanium seemed to be the most suitable, overcovering the area of resection and allowing correction of the resection area. In the case of other biomaterials, the exact match of the resection area and the implant is more challenging. However, several studies have shown good results after one-step bone lesions resection and cranioplasty (8, 19).

Another important issue is the tumor nature. As we mentioned above, single-stage cranioplasty with a template is usually performed for benign tumors since the surgical resection is potentially curative. However, this method can be used for malignant tumors (20). In this, it must be mentioned that (1) the time between CT scanning, cranioplasty manufacturing, and performing surgery should be minimized due to interim growth of the tumor; (2) presence of malignant tumors may require further radiation therapy; therefore, the implants material should be chosen to prevent radiation ulcers. Considering this, titanium does not seem to be the best choice due to high backscatter radiation to the scalp among other materials (21).

The limitation of our study is the small number of patients. However, skull lesions are not frequent, and reconstruction in the fronto-orbital region is not a surgery performed often. Including for this reason, up to date, there is no consensus on the optimal approach for simultaneous tumor resection and cranioplasty based on virtual surgical planning. The approaches differ by the modeling and manufacturing technologies and materials used. The general problem is difficulties in the evaluation of the cosmetic result. In our study, we based on the feelings of the patients themselves.

## Conclusion

Simultaneous resection and CAD/CAM titanium cranioplasty in the case of benign bone tumors in the fronto-orbital region is also a promising technique, with the aim of minimizing operation time and achieving a good esthetic outcome. Using a resection template allows to perform a practicable and precise craniotomy; however, in the case of large tumors with compound contours, it can be difficult. Unfortunately, there are a limited number of studies reproducing this approach with individualities. Future studies including more patients are needed for the standardization of this technique.

## Data Availability Statement

The raw data supporting the conclusions of this article will be made available by the authors, without undue reservation.

## Ethics Statement

Written informed consent was obtained from the individuals for the publication of any potentially identifiable images or data included in this article.

## Author Contributions

KY, IM, and SM: study concept and design. KY, AE, MO, MK, and MR: data acquisition and quality control of data. KY and IM: data analysis and interpretation. KY and AE: manuscript preparation. IM and SM: manuscript review. All authors contributed to the article and approved the submitted version.

## Funding

This study was supported by the state assignment (Theme No. 121030100312-0).

## Conflict of Interest

The authors declare that the research was conducted in the absence of any commercial or financial relationships that could be construed as a potential conflict of interest.

## Publisher's Note

All claims expressed in this article are solely those of the authors and do not necessarily represent those of their affiliated organizations, or those of the publisher, the editors and the reviewers. Any product that may be evaluated in this article, or claim that may be made by its manufacturer, is not guaranteed or endorsed by the publisher.
